# Olaparib Outcomes in Patients with BRCA 1-2 Mutated, Platinum-Sensitive, Recurrent Ovarian Cancer in Croatia: A Retrospective Noninterventional Study

**DOI:** 10.1155/2020/6423936

**Published:** 2020-06-20

**Authors:** Ana Majić, Branka Petrić Miše, Višnja Matković, Ingrid Belac Lovasić, Kristina Katić, Ivana Canjko, Ana Frobe, Žarko Bajić, Eduard Vrdoljak

**Affiliations:** ^1^Department of Oncology, University Hospital Center Split, School of Medicine, University of Split, Spinčićeva 1, Split HR-21.000, Croatia; ^2^Department of Gynecologic Oncology, University Hospital Center Zagreb, Petrova 13, Zagreb HR-10.000, Croatia; ^3^Department of Radiotherapy and Oncology, University Hospital Center Rijeka, Krešimirova 42, Rijeka HR-51.000, Croatia; ^4^Department of Radiotherapy and Oncology, University Hospital Center Osijek, Josipa Huttlera 4, Osijek HR-31.000, Croatia; ^5^Department of Oncology and Nuclear Medicine, University Hospital Center Sestre Milosrdnice, School of Dental Medicine, University of Zagreb, Zagreb HR-10.000, Croatia; ^6^Scientific Unit “Dr. Mirko Grmek”, Psychiatric Hospital “Sveti Ivan”, Jankomir 11, Zagreb HR-10.090, Croatia

## Abstract

Our objective was to assess the safety and efficacy of olaparib in maintenance therapy of BRCA 1-2 mutated, platinum-sensitive, recurrent ovarian carcinoma after the partial or complete response to the second or further lines platinum-based chemotherapy in a real-world setting. We performed a multicenter, real-world observational population-based cohort study on the whole population of Croatian patients initiated to olaparib maintenance therapy between 2016 and 2020. The primary endpoints were progression-free survival and the discontinuation of treatment because of adverse events. We enrolled the total population of 69 patients with the median (interquartile range; IQR) age of 53 (48–59), 56 (81%) of them with BRCA1 mutation. The median (IQR) follow-up was 16 (9–25) months. Treatment had to be discontinued because of toxicity in 2 (3%) and temporarily interrupted in 14 (20%), while dose was reduced because of toxicity in 18 (26%) of patients. Toxicity of any grade was observed in 61 (88%) patients and toxicity of grade 3 or 4 in 12 (17%). Median progression-free survival was 21 (95% CI 16-not calculable) months from the introduction of olaparib, and the median overall survival was not reached. Our study confirmed efficacy and safety of olaparib as the maintenance therapy of BRCA 1-2 mutated, platinum-sensitive, recurrent ovarian carcinoma. We observed the real-world efficacy and safety comparable to those observed in the randomized controlled trials. We found the interesting observation of better efficacy of 300 mg tablets, compared to 400 mg capsules, an issue that should be addressed on much larger real-world populations.

## 1. Introduction

Ovarian cancer is the most lethal gynecologic cancer, responsible for approximately 140,000 deaths in the world annually [1]. Unfortunately, there were no significant breakthroughs regarding overall survival in the therapy of ovarian cancer since introduction of platinum and paclitaxel as a standard treatment regimen. Recently, the incorporation of olaparib, niraparib, and rucaparib, PARP inhibitors, as maintenance therapy after response to a platinum-based therapy significantly changed the progression-free survival, increased response rate, and induced the long-term responses never seen before, especially in patients with BRCA mutated tumors [2–8]. The approval of three PARP inhibitors in rapid succession has resulted in a paradigm shift in the management of recurrent ovarian cancer. All three PARP inhibitors are administered orally [2–8]. An oral agent could be difficult to give for a patient with a recent history of small bowel obstruction and/or extensive peritoneal disease or preexisting refractory nausea, because of the likelihood of impaired drug absorption. Olaparib, previously required for proportion of patients demanding 16 capsules per day, was switched recently to a more manageable dosing regimen of 4 tablets per day [2, 3]. Now being phased out, the capsules are not interchangeable with tablets.

Randomized, controlled trials cannot always predict drug performance in real-world settings [9]. Consequently, observational studies and retrospective analyses are needed in order to evaluate the effects of anticancer therapies in broader, everyday cancer populations. Specifically, everyday cancer patients may not always mirror the characteristics of the patients treated in a study because of specific enrollment criteria, study-related procedures, ethical dilemmas, and differences inherent to those patients who chose to participate in clinical trials. Potential discrepancies, in both new drug efficacy and toxicity, between the results published in clinical trials and the results obtained from everyday clinical practice could be due to differences in patient selection, organizational issues, or multidisciplinary use, as well as general level of oncological care [9–11]. Consequently, results from randomized phase III trials are often difficult to be repeated in general clinical practice [9]. For example, survival of men with metastatic castrate-resistant prostate cancer treated with docetaxel and prednisone in routine practice was significantly shorter than for men included in clinical trials and was associated with more toxicity [9]. Based on that, strong recommendation should be made that all new drugs and treatment should be reviewed regarding their clinical benefit, in terms of retrospective analysis in different setups, countries, or healthcare systems. Large randomized phase III trials are performed in selective centers, with certain level of excellence in oncology care, potentially significantly higher than what the case is in an average oncology unit. Whilst majority of published articles are for phases I, II, and III trials, our knowledge of the real impact of new drugs on outcomes in real life patients finds these questionable [10, 11]. Consequently, retrospective analyses, phase IV observational clinical studies, and good cancer registries, institutional or even better country or region based, are essential to define the real impact of new therapies on our patients and healthcare systems. Poor adherence and quality of execution of diagnostic tests in oncology, especially complicated and expensive, are also potential reason for nonoptimal results of oncology care. BRCA testing is demanding from technical and time point of view and could be a potential reason for suboptimal penetration of PARP inhibitors in some oncology systems. Therefore, we performed a retrospective noninterventional study of diagnostic and treatment patterns and outcomes of patients with BRCA 1-2 mutated, platinum-sensitive, recurrent, ovarian carcinoma in Croatia. The objectives of our study were to investigate olaparib efficacy and toxicity in the total population of Croatian patients.

## 2. Methods

### 2.1. Study Design

We performed a multicenter, real-world retrospective observational population-based cohort study on the population of all Croatian patients diagnosed with BRCA 1-2 mutated recurrent, platinum-sensitive ovarian carcinoma and initiated to olaparib maintenance therapy between April 18, 2016, and January 4, 2020. Ethics committees of five participating institutions that cover the entire country population approved the protocol. We obtained the informed consents from all patients that were alive and accessible at the time of the data collection. We anonymized the data file before the analysis and performed the study in accordance with the World Medical Association Declaration of Helsinki of 1975 as revised in 2013 [12]. We had not pre-registered the study protocol, or reviewed the data centrally. The study was sponsored by AstraZeneca.

### 2.2. Study Population

The targeted population was patients diagnosed with BRCA 1-2 mutated, second or further lines, platinum-sensitive ovarian carcinoma treated with maintenance olaparib.

### 2.3. Sample Type and a Needed Sample Size

We assessed the entire population, so we have not selected the sample. We did not perform the power analysis before the study start.

### 2.4. Endpoints

The primary endpoint was the proportion of patients receiving at least one dose of olaparib, whose treatment was discontinued because of toxicity. Secondary safety endpoints were proportion of patients whose olaparib dose was reduced because of toxicity, incidence of treatment related adverse events of any grade, and of grade 3 or 4 according to Common Terminology Criteria for Adverse Events v4.0. The primary efficacy endpoint was a progression-free survival defined as the time in months from the initiation of olaparib to the date of progression, relapse, or death from any cause in patients who achieved a complete or partial response to the previous, second or further lines, platinum-based chemotherapy. Secondary efficacy endpoints were objective response rate defined as the complete response or partial response according to the RECIST version 1.1 for patients on olaparib not responding completely on previous platinum-based chemotherapy, disease control rate defined as the partial or complete response or the stable disease after the maintenance therapy with olaparib, and the overall survival, defined as the time in months from the initiation of olaparib to the death from any cause.

### 2.5. Treatment

Olaparib was administered in tablets or capsules twice daily until disease progression, unacceptable toxicity, or patient refusal.

### 2.6. Statistical Analysis

We performed the main safety analysis in the intention-to-treat population, and we used the Kaplan-Meier method to estimate the median progression-free survival and overall survival with 95% confidence intervals (CI) in the population who received at least one dose of olaparib. In the exploratory analysis, we used the Cox proportional hazards regression, with Efron method to handle ties, to estimate the hazard ratios (HR) for progression and the binary logistic regression to estimate the odds for adverse events in different patients subgroups. We check the proportional hazard assumption by assessing the nonzero slopes of the generalized linear regression of the scaled Schoenfeld residuals on row and on the log-time, and by visual inspection of the parallelism and closeness of Kaplan–Meier curve observed, and the survival curves predicted by the Cox regression as well as of log-log survival plots of the two different patients groups. We controlled the false positive rate using the Benjamini–Hochberg procedure with the false discovery rate set in advance at FDR<10%. We set two-tailed statistical significance at *p* < 0.05 and calculated all confidence intervals (CI) at 95% level. We performed the statistical data analysis using StataCorp 2019 (Stata Statistical Software: Release 16. College Station, TX: StataCorp LLC).

## 3. Results

### 3.1. Patients Characteristics

Since April 18, 2016, a total of 69 patients with known BRCA 1-2 mutation were treated with second or later lines of platinum-based chemotherapy for recurrent ovarian carcinoma, have achieved partial or complete response, and have received olaparib maintenance therapy in the Republic of Croatia. Median (IQR) age at diagnosis was 53 (48–59) years ranging from 35 to 81 years (Table 1). Majority of patients had BRCA 1 mutation type (56) (81%) and in over two-thirds it was determined by the blood test. Olaparib was initiated in the median (IQR) 36 (26–63) months from the primary surgery, after the median of two previous chemotherapy lines (Table 2). Median (IQR) follow-up was 16 (9–25) months from the introduction of olaparib, ranging from 1 to 41 months, and the median (IQR) number of olaparib cycles was 11 (6–18).

### 3.2. Safety

Treatment had to be discontinued because of toxicity in 2 (3%) patients. Treatment was temporarily interrupted in 14 (20%) patients. Toxicity of any grade was observed in 61 (88%) patients, and toxicity of grade 3 or 4 in 12 (17%) (Table 3). We have not observed grade 5 adverse events in any patient. Hematologic toxicity was observed in 35 (51%) and nonhematologic in 54 (78%) patients. There were 28 (41%) patients with both hematologic and nonhematologic adverse events of any grade. Olaparib dose was reduced because of toxicity in 18 (26%) patients. In the exploratory analysis, we found that patients with significantly higher odds for dose reduction were those with ECOG status 1 before olaparib (OR = 4.00; 95% CI 1.16 to 13.8; *p*=0.028; FDR ≤ 10%). Age at diagnosis, primary tumor location, histological type, grade, FIGO stage, macroscopic residual disease after the primary surgery, comorbidities, body mass index, number of chemotherapy lines before olaparib, response to the previous chemotherapy, and olaparib formulation were not significant bivariable predictors of the dose reduction because of toxicity. ECOG status before olaparib remained significant predictor of the dose reduction after the adjustment for all these variables using a multivariable binary logistic regression (OR = 4.84; 95% CI 1.02–23.0; *p*=0.047; FDR < 10%).

### 3.3. Efficacy

**Figure 1:**
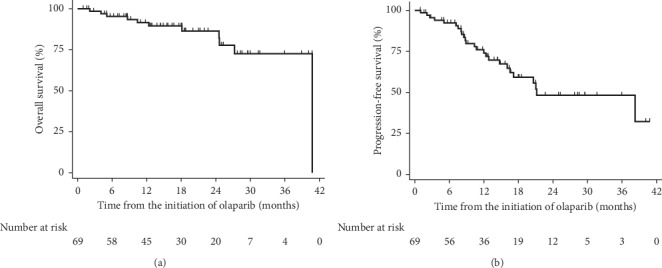
Kaplan–Meier curves of the overall and progression-free survival from the initiation of olaparib (*n* = 69).

Median progression-free survival was 21 (95% CI 16-not calculable) months from the introduction of olaparib, and the median overall survival was not reached (Figure 1). Out of 44 patients with the partial response to the previous chemotherapy, none experienced the complete response to the maintenance therapy with olaparib, and 5/44 (11%) experienced the partial response (Table 2). In the exploratory analysis, we observed the significantly lower hazard for progression in patients who experienced any toxicity (HR = 0.11; 95% CI 0.04–0.32; *p* < 0.001; FDR<10%), and the significantly lower hazard for progression in patients treated with 300 mg tablets, than in those treated with 400 mg capsule (HR = 0.28; 95% CI 0.10–0.82; *p*=0.020; FDR<10%). Median progression-free survival in patients on capsule was 17 (95% CI 10–38) months, while in patients treated with tablets the median progression-free survival was not reached. The difference between capsule and tablets remained significant and clinically relevant after the adjustment for response to the last previous treatment, age at diagnosis, primary tumor location, FIGO stage, ECOG status before the initiation of olaparib, BMI, BRCA mutation type, comorbidities, previous chemotherapy lines, and as the time-dependent covariate number of olaparib maintenance therapy cycles by Cox regression (HR = 0.17; 95% CI 0.04–0.74; *p*=0.018; FDR<10%).

## 4. Discussion

Analyzing the total population of patients initiated on olaparib maintenance therapy in Croatia between April 18, 2016, and January 4, 2020, we observed the real-world olaparib maintenance therapy efficacy and safety comparable to the ones observed in the randomized controlled trials [2, 3, 13]. The progression-free survival (21 months) was longer than in the comparable Italian analysis of 234 BRCA 1-2 mutated patients who received olaparib in 13 Italian centers between September 1 2015 and May 31 2019 (14.7 months) [14], but almost the same as in the SOLO2 multicenter, double-blind, randomized, placebo-controlled, phase 3 trial (19.1 months) [3]. Italian authors explained this lower progression-free survival in their study by its real-world design and the more selected patients, highly experienced therapy providers with more strict rules and schedules in SOLO2. Although this explanation is plausible, we think that the difference between Italian real-world study and SOLO2 results may at least partially be further explained by the differences in the inclusion criterion and the olaparib administration. Namely, in Italian study 37 (16%) patients had stable or progressive disease after the last previous treatment or the response was unknown, while in SOLO2, as in our study, all patients had objective (complete or partial) response before the introduction of olaparib. So, the Italian study enrolled patients with the worse response to the previous treatment and this could partially cause the lower progression-free survival later on. Another potentially important difference was in the administration of olaparib. In Italian study, patients were initiated to 400 mg capsule formulation, while in SOLO2 patients received 300 mg tablets. Tablets and capsules have different pharmacokinetic properties and are not bioequivalent and interchangeable [15]. Tablets have better bioavailability and Mateo et al. adaptive, phase 1 trial showed that the steady-state maximum plasma concentration and the area under the plasma concentration-time curve are higher in tablets of 250 mg than in capsules of 400 mg and that the patients' exposure after the 300 mg tablets even exceeded the exposure of 400 mg capsule [16]. Another point to be stated here is greater probability of appropriate compliance of tablets over capsules simply due to the number, 4 tablets over 16 capsules. Our finding of better efficacy of tablets after the adjustment for many relevant covariates further indicated noncomparability of two formulations. Tablets instead of capsules are probably part of the explanation for markedly longer progression-free survival in SOLO2 [3] and our study compared to Study 19 (11.2 months in patients with BRCA 1-2 mutation) [13]. Progression-free survival in the Italian study was almost the same as in the real-world data study performed in Korea in 2016–2018 [17]. Our previous explanation for somewhat shorter progression-free survival in the case of Italian study which postulated the effect of 16% of patients with no objective response to the previous chemotherapy does not hold for the Korean study as they enrolled only the patients with complete or partial response to the last platinum-based treatment before initiation of olaparib, as we and SOLO2 did. But as in the case of Italian study, they administered olaparib in 400 mg capsules, which may further strengthen our interpretation that postulated better efficacy of 300 mg tablets formulation. This is yet another indication that the problem of capsules and tablets efficacy, and not only the safety, should be further assessed using the properly powered real-world analysis, because randomized controlled trial is highly unlikely due to commercial reasons. Although a real-world study performed in Switzerland and France does not explicate the formulation used, the fact that it enrolled the patients from 2014 to 2018 indicates the predominant usage of capsule formulation [18]. If so, this may, as above, at least partially explain the progression-free survival of 12.7 months with the overall follow-up of 21 months. The overall survival of 35.4 months in this European study was comparable to overall survival observed in Study 19 [2], and the real-world study performed in Sweden on the registry data (33 months) [19].

Discontinuation of olaparib maintenance therapy was low in our study (3%) and completely comparable to the results of other studies. It was almost the same as in Study 19 (2%) [2], the real-world study conducted in France and Switzerland (4%) [18], Italy (5%) [14], and Korea (4%) [17]. Somewhat larger number of patients whose therapy with olaparib was discontinued because of adverse events in SOLO2 (11%) was already plausibly explained with the relatively longer follow-up (21 months) [3]. Percentage of patients whose olaparib dose was reduced to control the adverse events has been very similar across different studies as well, ranging from 14% in Swedish registry real-world study [19], over 21%, 23%, 25%, and 26% in Italian study [14], Study 19 [2], SOLO2 [3], and our study, respectively, to 36% in Korean study [17]. It seems that the incidence of adverse events is somewhat higher in randomized controlled trials (35% and 36% in Study 19 and SOLO2, respectively) than in real-world data studies (17% in our study and 17% in Chinese study [20]). This should probably be explained by the more rigorous documentation and adverse events detection protocols used in randomized controlled trials than in the routine everyday clinical practice.

The main limitations of our study were relatively short overall follow-up period from the initiation of olaparib and nonexistence of the randomized control group particularly in the exploratory analysis of the differences in progression-free survival between two formulations.

## 5. Conclusions

Our study indicated the good efficacy and safety of olaparib as the maintenance therapy of BRCA 1-2 mutated platinum-sensitive, recurrent ovarian carcinoma. We observed the real-world efficacy and safety comparable to those observed in the randomized controlled trials. We found the interesting observation of better efficacy of 300 mg tablets, compared to 400 mg capsules, and this issue should be further addressed with a properly powered real-world data analysis.

## Figures and Tables

**Table 1:** Characteristics of patients before the introduction of olaparib (*n* = 69).

	*n* (%)
Age at diagnosis (years), median (IQR)	53 (48–59)
ECOG performance status at diagnosis		
0	53 (77)
1	16 (23)
Primary tumor location		
Ovarian	49 (71)
Fallopian tube	15 (22)
Peritoneal	5 (7)
Histological type		
Serous	66 (96)
Endometrial	2 (3)
Mucinous	1 (1)
Gradus III	65 (94)
FIGO stage at diagnosis		
I	4 (6)
II	16 (23)
III	38 (55)
IV	10 (15)
Unknown	1 (1)
BRCA mutation type		
BRCA 1	56 (81)
BRCA 2	13 (19)
Both	0 (0)
Testing for BRCA		
Tumor	23 (34)
Blood	44 (65)
Both	1 (1)
Comorbidities	28 (41)
Residual disease after primary surgery		
No macroscopic disease	27 (39)
Macroscopic disease	33 (48)
Unknown	9 (13)
Ascites		
No	35 (51)
Yes	26 (38)
Unknown	8 (12)

Data are presented as number (percentage) of patients if not stated otherwise. IQR = interquartile range. Data were missing for BRCA testing for 1 (1%) patient.

**Table 2:** Introduction of olaparib, maintenance therapy, and efficacy outcomes (*n* = 69).

	*n* (%)
Time from surgery (months), median (IQR)	36 (26–63)
Chemotherapy lines before, median (IQR)	2 (2-3)
Chemotherapy lines before		
2	38 (55)
3	19 (28)
4	6 (9)
5	4 (6)
≥6	2 (3)
Chemotherapy protocol before		
Gemcitabine/carboplatin	10 (16)
Paclitaxel/carboplatin	45 (74)
Mono carboplatin	4 (7)
Other platin-based chemotherapy	2 (3)
Response to the chemotherapy before		
Complete response	25 (36)
Partial response	44 (64)
ECOG performance status before	
0	55 (80)
1	14 (20)
Formulation		
Tablet	35 (51)
Capsule	34 (49)
Cycles, median (IQR)	11 (6–18)
Duration (months)	11 (7–18)
Outcomes (*n* = 44)^*∗*^		
Complete response	0 (0)
Partial response	5 (11)
Stable disease	17 (39)
Progressive disease	22 (50)
Objective response rate	5 (11)
Disease control rate	22 (50)

Data are presented as number (percentage) of patients if not stated otherwise. IQR = interquartile range. Data were missing for chemotherapy protocol before introduction of olaparib for 8 (12%) patients. ^*∗*^Efficacy outcomes were presented only for patients who had partial response to the previous chemotherapy.

**Table 3:** Safety outcomes.

	Any grade		Grades 3-4	
	*n* (%)	(95% CI)	*n* (%)	(95% CI)
Any toxicity	61 (88)	(78–95)	12 (17)	(9–28)
Hematologic toxicity	35 (51)	(38–63)	12 (17)	(9–28)
Anemia	29 (42)	(30–55)	9 (13)	(6–23)
Neutropenia	14 (20)	(12–32)	3 (4)	(1–12)
Elevation of creatinine	10 (14)	(7–25)	1 (1)	(0–8)
Thrombocytopenia	4 (6)	(2–14)	3 (4)	(1–12)
Nonhematologic toxicity	54 (78)	(67–87)	0 (0)	(0–5)
Fatigue	48 (70)	(57–80)	0 (0)	(0–5)
Nausea	31 (45)	(33–57)	0 (0)	(0–5)
Constipation	4 (6)	(2–14)	0 (0)	(0–5)
Vomit	3 (4)	(1–12)	0 (0)	(0–5)
Diarrhea	3 (4)	(1–12)	0 (0)	(0–5)
Artalgia	3 (4)	(1–12)	0 (0)	(0–5)
Abdominal pain	2 (3)	(0–10)	0 (0)	(0–5)

## Data Availability

Data are available upon request to the corresponding author.

## References

[1] Bray F., Ferlay J., Soerjomataram I., Siegel R. L., Torre L. A., Jemal A. (2018). Global cancer statistics 2018: GLOBOCAN estimates of incidence and mortality worldwide for 36 cancers in 185 countries. *CA: A Cancer Journal for Clinicians*.

[2] Ledermann J., Harter P., Gourley C. (2012). Olaparib maintenance therapy in platinum-sensitive relapsed ovarian cancer. *New England Journal of Medicine*.

[3] Pujade-Lauraine E., Ledermann J. A., Selle F. (2017). Olaparib tablets as maintenance therapy in patients with platinum-sensitive, relapsed ovarian cancer and a BRCA1/2 mutation (SOLO2/ENGOT-Ov21): a double-blind, randomised, placebo-controlled, phase 3 trial. *Lancet Oncology*.

[4] Mirza M. R., Monk B. J., Herrstedt J. (2016). Niraparib maintenance therapy in platinum-sensitive, recurrent ovarian cancer. *New England Journal of Medicine*.

[5] Coleman R. L., Oza A. M., Lorusso D. (2017). Rucaparib maintenance treatment for recurrent ovarian carcinoma after response to platinum therapy (ARIEL3): a randomised, double-blind, placebo-controlled, phase 3 trial. *Lancet*.

[6] Pilié P. G., Tang C., Mills G. B., Yap T. A. (2019). State-of-the-art strategies for targeting the DNA damage response in cancer. *Nature Reviews Clinical Oncology*.

[7] Musella A., Bardhi E., Marchetti C. (2018). Rucaparib: an emerging parp inhibitor for treatment of recurrent ovarian cancer. *Cancer Treatment Reviews*.

[8] Tomao F., Bardhi E., Di Pinto A. (2019). Parp inhibitors as maintenance treatment in platinum sensitive recurrent ovarian cancer: an updated meta-analysis of randomized clinical trials according to BRCA mutational status. *Cancer Treatment Reviews*.

[9] Templeton A. J., Vera-Badillo F. E., Wang L. (2013). Translating clinical trials to clinical practice: outcomes of men with metastatic castration resistant prostate cancer treated with docetaxel and prednisone in and out of clinical trials. *Annals of Oncology*.

[10] Sargent D. (2010). What constitutes reasonable evidence of efficacy and effectiveness to guide oncology treatment decisions?. *The Oncologist*.

[11] George S. L. (1996). Reducing patient eligibility criteria in cancer clinical trials. *Journal of Clinical Oncology*.

[12] World Medical Association (2013). World Medical Association Declaration of Helsinki: ethical principles for medical research involving human subjects. *JAMA*.

[13] Ledermann J., Harter P., Gourley C. (2014). Olaparib maintenance therapy in patients with platinum-sensitive relapsed serous ovarian cancer: a preplanned retrospective analysis of outcomes by BRCA status in a randomised phase 2 trial. *The Lancet Oncology*.

[14] Cecere S. C., Giannone G., Salutari V. (2020). Olaparib as maintenance therapy in patients with BRCA 1-2 mutated recurrent platinum sensitive ovarian cancer: real world data and post progression outcome. *Gynecologic Oncology*.

[15] Perego G., Nozza R., Oggionni E. (2020). Pharmacological issues concerning olaparib capsule and tablet formulations in treating ovarian cancer: are they really the same drug?. *Journal of Oncology Pharmacy Practice*.

[16] Mateo J., Moreno V., Gupta A. (2016). An adaptive study to determine the optimal dose of the tablet formulation of the PARP inhibitor olaparib. *Targeted Oncology*.

[17] Paik E. S., Lee Y. J., Lee J.-Y. (2019). Real-world experience of olaparib maintenance in high-grade serous recurrent ovarian cancer patients with BRCA1/2 mutation: a Korean multicenter study. *Journal of Clinical Medicine*.

[18] Labidi-Galy S. I., de La Motte Rouge T., Derbel O. (2019). Clinical factors associated with prolonged response and survival under olaparib as maintenance therapy in BRCA mutated ovarian cancers. *Gynecologic Oncology*.

[19] Eriksson I., Wettermark B., Bergfeldt K. (2018). Real-world use and outcomes of olaparib: a population-based cohort study. *Targeted Oncology*.

[20] Ni J., Cheng X., Zhou R. (2019). Olaparib in the therapy of advanced ovarian cancer: first real world experiences in safety and efficacy from China. *Journal of Ovarian Research*.

